# Case of Eosinophilic Cystitis Treated with Suplatast Tosilate as Maintenance Therapy

**DOI:** 10.1155/2012/354219

**Published:** 2012-09-11

**Authors:** Tateki Yoshino, Hiroyuki Moriyama

**Affiliations:** Department of Urology, JA Onomichi General Hospital, Hiroshima 722-0018, Japan

## Abstract

Eosinophilic cystitis is a rare inflammatory lesion of the bladder, characterized by massive eosinophilic infiltration of the bladder wall. Its cause is not known definitely. A 49-year-old man consulted our department with a miction pain, gross hematuria, and frequent micturition. Urinalysis showed combined hematuria and pyuria, but urine culture was sterile. Abnormal findings of laboratory examination included an elevated white blood cell (WBC) count (15,700/**μ**L) and the proportion of eosinophils in the peripheral blood was 12% of the WBCs (normal 0–5%). Cystoscopy revealed a solid mass with severe edematous mucosa. Magnetic resonance imaging (MRI) also indicated marked bladder wall thickening, which was suspected for invasive bladder cancer. Transurethral biopsy of the bladder mass was performed with pathological examination revealing features of eosinophilic cystitis. After administration of a combination of prednisolone and suplatast tosilate, followed by monotherapy with suplatast tosilate, regression of the bladder mass, and normalization of the count of peripheral eosinophils were achieved. Fourteen months after steroid therapy, under treatment with suplatast tosilate, there was no relapse of urinary symptoms and the bladder mass.

## 1. Introduction

Eosinophilic cystitis is a rare inflammatory lesion of the bladder of unknown origin. The majority of knowledge about this disorder is based on the case reports. Brown [1] and Palubinskas [2] first reported the disease in 1960. Not frequently, the disease mimic the bladder cancer with clinical, radiographic, and cystoscopic features. The definitive diagnosis can be made only by histopathological examination of biopsy specimens. In this report, we present a case of eosinophilic cystitis mimicking invasive bladder cancer treated with prednisolone and suplatast tosilate.

To our knowledge, this is the first case of eosinophilic cystitis treated with suplatast tosilate in the English literature.

## 2. Case Report 

A 49-year-old man consulted our department with a miction pain, gross hematuria, and frequent micturition. His past medical history included none. He had experienced no signs of rheumatoid disease, bronchial asthma, or other known allergic disease and had received no medication. There was no history of urinary symptoms and urinary tract infection. The results of physical examination were unremarkable.

Urinalysis showed combined hematuria and pyuria, but urine culture was sterile. Urine cytology indicated atypical cells. Abnormal findings of laboratory examination included an elevated WBC count (15,700/*μ*L) and the proportion of eosinophils in the peripheral blood was 12% of the WBCs (normal 0–5%). Immunoglobulin analysis showed normal range of IgE (9.8 IU/mL, normal < 173 IU/mL). In spite of administration of antibiotics, his symptoms and urinalysis result did not improved.

**Figure 1:**
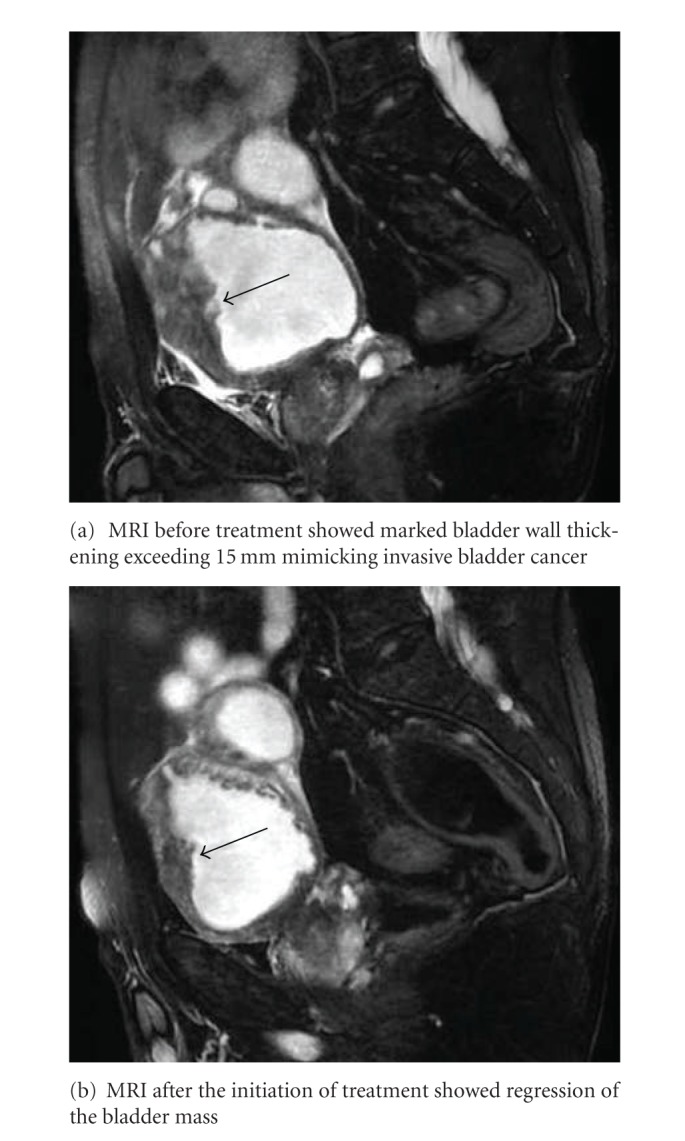
MRI findings (sagittal section). Arrow; bladder wall thickening.

Cystoscopy revealed a solid mass covered with clots at the dome to anterior wall and several diverticula of the bladder with severe edematous mucosa. Contrast-enhanced MRI also indicated marked bladder wall thickening exceeding 15 mm (Figure 1(a)), which was suspected for invasive bladder cancer. 

**Figure 2:**
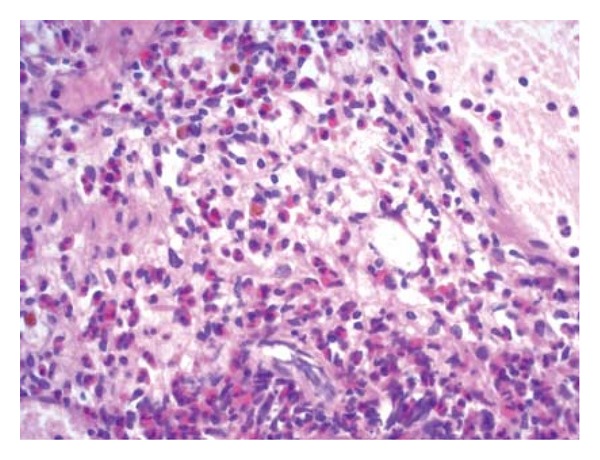
Microscopic appearance of the bladder wall with massive infiltration of eosinophils into the muscular layer.

Transurethral biopsy of the bladder mass was performed with pathological examination revealing features of eosinophilic cystitis, infiltration of abundant eosinophils from the submucosa to the muscular layer being evident (Figure 2). 

The patient was given a combination of prednisolone 30 mg per day with a tapering course for 8 weeks and suplatast tosilate, followed by monotherapy with suplatast tosilate. After treatment, regression of the bladder mass (Figure 1(b)), and normalization of the count of peripheral eosinophils (2.7% of the WBCs) were achieved. In addition, cystoscopic findings two months after the initiation of treatment indicated the improvement of bladder mass and edematous mucosa.

Fourteen months after steroid therapy, under treatment with suplatast tosilate, there was no relapse of urinary symptoms and the bladder mass.

## 3. Discussion 

Eosinophilic cystitis is a rare condition characterized histologically by eosinophilic infiltration of the submucosa and muscular layer. It is usually associated with marked irritative lower urinary tract symptoms, such as dysuria, frequent micturition, and miction pain. Eosinophilic cystitis affects men slightly more often than women, with a male-to-female ratio of 1.3 : 1 [3]. The etiology of eosinophilic cystitis remains unknown, but it is believed to be associated with injury; drugs (e.g., methicillin, warfarin, anthranilic acid, intravesical mitomycin, and thiotepa); bacterial, viral, and parasitic infections; and reactions to food (e.g., vegetables, spices, and chocolate), and other allergens. Allergic reactions have been suggested to be predisposing factors in the pathogenesis and food allergy, and hypersensitivity to inhalant allergens and drugs have been discussed in relation to urinary tract lesions. It has been reported that peripheral eosinophilia is present in 43% of patients with eosinophilic cystitis, but elevated serum IgE is uncommon [4], and their interrelations are unclear. In the past report [5], urinalyses of 77% of patients showed hematuria and/or pyuria. The ratio of positive urine culture was only 20.5%. 

Radiographic findings may show variable thickening of the bladder wall, ranging from diffuse thickening to mass formation. The pseudotumorous form of eosinophilic cystitis is characterized by an extensive infiltration of the bladder wall that may resemble an invasive tumor [6].

Diagnosis can be made by histological examination of a biopsy specimen of the bladder. Histologically, the submucosa is edematous, containing a mixed inflammatory infiltrate in which eosinophils are prominent. Eosinophilic infiltration may be the final pathway by which endogenous and exogenous allergens cause IgE-mediated degranulation of mast cells and the release of eosinophilic chemotactic factors, such as various cytokines. Additionally, previous studies suggested that activated eosinophils released cytotoxic cationic proteins that could induce tissue damage [7]. These mechanisms were speculated for primary disease process of eosinophilic cystitis [8].

While treatment for eosinophilic cystitis has not yet been standardized, it was reported as follows: (1) removal of the allergen or discontinuation of drug when an allergy is the clear cause; (2) administration of an antihistaminic agent and NSAIDs; (3) administration of steroids; (4) transurethral resection of the disorder lesion; or (5) administration of immunosuppressive agents, such as cyclosporine. Because of probable immunologic nature of disease, corticosteroids have become the mainstay of the treatment, especially in patients who have an unresolved disease with anti-inflammatory drugs and antihistamines and have wide tumoral lesions.

In the present case, because oral suplatast tosilate was effective for eosinophilic cystitis, prednisolone was tapered gradually and withdrawn. To our knowledge, this is the first case of eosinophilic cystitis treated with suplatast tosilate in the English literature. Previous Japanese literature have also reported the efficacy of suplatast tosilate for eosinophilic cystitis [9]. Suplatast tosilate was widely known as a therapeutic agent for bronchial asthma, atopic dermatitis, and allergic rhinitis. In action mechanisms of suplatast tosilate, it inhibits the production of helper T-cell-derived interleukin 4 and 5 (IL-4 and IL-5), suppressing the production of IgE from B cells and cytokines from mast cells [10]. In addition, low production of IL-5 leads to the inhibition of eosinophil differentiation and activation, and further, the release of cytotoxic cationic proteins which can induce tissue damage [11]. Thinking of primary disease process of eosinophilic cystitis, it was regarded that suplatast tosilate is efficacious for this disorder from its action mechanisms. 

Only in patients who have a disease intractable to conservative treatments as previously mentioned and presenting as profuse hematuria, contracted bladder, uni- or bilateral distal ureteral involvement together with renal failure, partial or total cystectomy, and diversion must be considered [3, 4, 12].
